# Registry of Senior Australians (ROSA) Outcome Monitoring System: Using Linked Aged Care and Health Data to Monitor Long-Term Care Quality and Safety

**DOI:** 10.23889/ijpds.v11i4.3812

**Published:** 2026-07-06

**Authors:** Tesfahun C Eshetie, Gillian E Caughey, Catherine Lang, Chin Tho Leong, Laura Young, Maria C Inacio, on behalf of the ROSA Outcome Monitoring System Advisory Committee

**Affiliations:** 1 Registry of Senior Australians Research Centre, South Australian Health and Medical Research Institute, Adelaide, Australia; 2 Registry of Senior Australians Research Centre, Caring Futures Institute, College of Nursing and Health Sciences, Flinders University, Adelaide, Australia; 3 UniSA Allied Health and Human Performance, University of South Australia, Adelaide, Australia

## Background

The Registry of Senior Australians (ROSA) has developed a national system to monitor and benchmark the quality and safety of long-term care in residential facilities and home settings. The ROSA Outcome Monitoring System (OMS) examines the variation in the quality and safety of long-term care in Australia. This presentation introduces the ROSA OMS and highlights its pragmatic approach for long-term care quality and safety monitoring.

## Methods

The ROSA OMS employs linked national and state-based aged care and health care data collections. Non-Indigenous individuals aged ≥65 years or Aboriginal and Torres Strait Islander people aged ≥50 years receiving long-term care are included. Guided by the ROSA OMS Advisory Committee (28 members across 18 institutions, including aged care providers, peak bodies, clinicians and consumer representatives), the ROSA OMS examines the annual prevalence and variation in15 risk-adjusted quality and safety indicators of long-term care. It provides national and achievable benchmarks for providers, public national and provider-specific reports, and risk profiling tools to support risk reduction strategies.

## Results

The ROSA OMS has released individualised, direct-to-provider quality and safety monitoring reports (n=150 in 2022/2023, n=305 in 2024, and n=311 in 2025 for eligible long-term care providers) and biennial public national reports. Eleven risk prediction models have been developed to identify older people at risk of experiencing monitored indicators.

## Conclusion

ROSA OMS is an academic-, clinician-, and stakeholder-led national program generating high-quality evidence using routinely collected national and state-based data to drive improvements in long-term care quality and safety across Australia.

